# Christian religion and spirituality in eating disorder development, experience, and recovery: an exploration of lived experience in Australia and New Zealand

**DOI:** 10.3389/fpsyg.2026.1764418

**Published:** 2026-02-17

**Authors:** Hayley Thomas, Clare O’Callaghan, Megan Best, Michael Bräutigam, Thomas Kimber, Tracey Wade, Nancy Sturman

**Affiliations:** 1 General Practice Clinical Unit, Medical School, The University of Queensland, Herston, QLD, Australia; 2 Institute for Ethics and Society, The University of Notre Dame Australia, Broadway, NSW, Australia; 3 Centre for Theology and Psychology, Melbourne School of Theology, Wantirna, VIC, Australia; 4 Flinders Institute of Mental Health and Wellbeing, Flinders University, Bedford Park, SA, Australia

**Keywords:** attachment theory, Christianity, eating disorders, perfectionism, qualitative research, religion, spirituality

## Abstract

**Background:**

Eating disorders are complex conditions, with aetiological factors and impacts across multiple domains. Religion and spirituality are areas of individual diversity that may represent a source of strength or struggle for those experiencing mental illness. However, there is limited and mixed evidence regarding the influence of religion and spirituality in eating disorders, and this is not well integrated within healthcare.

**Aim:**

To explore the interplay between Christian religion and spirituality and eating disorder development, experience and recovery, from the perspectives of Australians and New Zealanders with lived experience.

**Methods:**

Qualitative methodology using reflexive thematic analysis and selected grounded theory techniques. Data is based on semi-structured interviews with 23 participants who had a Christian background and self-identified as having partially or fully recovered from an eating disorder. Trustworthiness was supported by investigator reflexivity and triangulation.

**Results:**

Analysis highlighted an overarching experience of eating disorders and Christian spirituality as two non-linear, interwoven journeys. Three themes and seven subthemes elucidated this dynamic. Themes included (1) “Not enough”—A sense of not being “good enough” and/or “safe enough” in an eating disorder could be exacerbated or alleviated by Christian religion and spirituality; (2) “Wrestling”—Eating disorders often triggered religious and spiritual, which could change spiritual trajectories; and (3) “Help to heal”—Some participants found comfort, hope, and a foundation for eating disorder recovery in their Christian religion and spirituality, often experienced as embracing God’s grace (that is, God’s unmerited love and favour).

**Conclusion:**

This study highlights previously under-recognised religious and spiritual influences upon protective and predisposing factors for eating disorders, including perfectionism, attachment and identity formation. Results could inform more personalised approaches to caring for Christians experiencing eating disorders. Findings also suggest a need for similar research with other religious and spiritual traditions.

## 1 Introduction

Eating disorders (EDs) are serious conditions that are rising in prevalence and require more effective approaches to care (Hay et al., 2023; Solmi et al., 2024). Globally, lifetime prevalence of EDs is estimated to be between 0.74 and 2.2% in males, and between 2.58 and 8.4% in females (Hay et al., 2023). EDs have a profound impact on those who experience them and their loved ones and carry a high societal cost (Fox et al., 2017; Hay et al., 2023). Fewer than half of patients with anorexia or bulimia nervosa fully recover, and anorexia nervosa has a mortality rate of approximately 5% (Steinhausen, 2002; Steinhausen and Weber, 2009). The complex nature of EDs is increasingly acknowledged, with well-recognised biological, psychological, developmental and sociocultural dimensions (Kaye et al., 2017). Potential religious and spiritual influences remain less well-understood, despite evidence suggesting that these may play a dual role in EDs, at times offering protection, while at others contributing to ED onset or persistence (Akrawi et al., 2015).

Religion and spirituality represent overlapping but distinct terms (Hill et al., 2000; Baumsteiger and Chenneville, 2015; de Brito Sena et al., 2021). Religion comprises a system of beliefs and practices related to the divine or transcendent that are shared by an adherent community (Koenig et al., 2023b). Spirituality is a more nebulous term, which in healthcare literature has been related to individuals’ sources of connection, meaning, purpose, well-being and inner peace (de Brito Sena et al., 2021).

Religion and spirituality touch on deep aspects of personal identity, experience and meaning. This makes their relevance to mental health unsurprising, a connection now supported by a significant body of evidence (Koenig, 2009; Koenig et al., 2023b). A substantial body of work has also identified religious and spiritual associations with conditions that are often comorbid with EDs, including depression, anxiety and substance-use disorders (Hambleton et al., 2022; Koenig et al., 2023b). Such evidence suggests a mixed and bidirectional influence of religion upon mental health, with most studies suggesting a positive influence, though some find an adverse effect (Koenig et al., 2023a). It has been suggested that these impacts are mediated both directly, through healthy or unhealthy coping behaviours, and indirectly, through influences on biopsychosocial determinants of mental health (Koenig et al., 2023a).

However, research concerning religious and spiritual aspects in EDs is limited. Historically, religious factors (most notably asceticism—that is, strict physical self-denial for religious reasons (Harich-Schwarzbauer et al., 2011)) have been associated with conditions resembling EDs, though the extent to which these reflect modern EDs is debated (Vandereycken and van Deth, 1994; Sukkar et al., 2017). More recent evidence regarding the impact of religion and spirituality in EDs suggests a mixed role, whereby some expressions of religion and spirituality may be protective, while others may increase ED risk (Akrawi et al., 2015; Richards et al., 2020). A systematic review of quantitative studies investigating religiosity and spirituality in relation to disordered eating and body image concerns concluded that their effect depended upon a person’s motivation for religious engagement, and the nature of their relationship with God (Akrawi et al., 2015). Specifically, deeply internalised religious belief and motivation (intrinsic religiosity) was associated with lower levels of disordered eating and body image concerns, whereas pursuit of religion for social reasons or personal comfort (extrinsic religiosity) was associated with increased levels. The review also found that religious angst (religious anxiety, doubt, and/or a sense of being punished by God) was associated with higher levels of disordered eating and body image concerns, as opposed to a secure relationship with God. Qualitative studies also suggest that people with EDs tend towards dualistic conceptions of God’s nature as caring and merciful, or punitive, distant, uninvolved and/or disciplinarian (Morgan et al., 2000; Marsden et al., 2007; Sosin, 2008; Mulvihill, 2009; Gothard, 2011). Additionally, people with lived experience of an ED have called for a more comprehensive concept of ED recovery beyond symptom remission (de Vos et al., 2017; Wetzler et al., 2020). Their concept of recovery resonates with current understandings of spirituality, touching on connection, identity, hope, meaning, purpose, empowerment, and self-compassion.

There is evidence that people with disordered eating often experience heightened religious and spiritual struggle (Exline et al., 2016) and low levels of spiritual wellbeing (Watkins et al., 2006; Wetherbe Hayman et al., 2007; Phillips et al., 2015). EDs tended to undermine patients’ preexisting spirituality and some withdrew from religious practices as a result (Graham et al., 1991; Abel, 2005). Participants in qualitative studies described a sense of spiritual disconnection, suffering and brokenness during their EDs, and some identified spiritual reconnection (religious or non-religious) as an important characteristic of recovery (Sosin, 2008; Matusek and Knudson, 2009; de Vos et al., 2017; Richards et al., 2018). During the process of recovery, some reported relinquishing previous religious practices, though in some cases retained a belief in a higher power and spiritual engagement (Marsden et al., 2007; Matusek and Knudson, 2009; Tramontana, 2009). Others turned to or resumed religious faith during their ED journey and viewed this as central to their healing (Hsu et al., 1992; Marsden et al., 2007; Matusek and Knudson, 2009).

This is consistent with Pargament’s conceptualisation of religious and spiritual journeys as a dynamic and evolving “search for the sacred” (Pargament, 2007). He suggests that when people with religious or spiritual beliefs are faced with a stressor (such as mental illness) that threatens or points to the limits of their understanding of the sacred, they usually engage in “conservational spiritual coping.” Such coping seeks to preserve the sacred and may be “positive,” such as through seeking stronger connection, love, care or help from God, or “negative,” such as experiencing feelings of being punished or abandoned by God, and a questioning of God’s love or power (Pargament, 2007, 2010). If the stressor overwhelms a person’s ability to cope, they may enter a period of spiritual turmoil, experiencing struggles that may be intrapsychic (e.g., doubts about faith, sense of guilt), interpersonal (e.g., feeling abandoned by or disagreeing with their faith community) and/or divine (e.g., feeling punished or abandoned by, or angry at, God). This leads either to a return to their former understanding, permanent disengagement from spirituality, temporary disengagement followed by rediscovery, or transformation in their understanding of the sacred (Pargament, 2007). Because spiritual struggle overlies and can influence the distress arising from mental illness, Pargament argues that it is important for those seeking to provide mental health support to be aware of these dynamics.

However, religious and spiritual aspects are rarely considered in mainstream ED treatment, although some self-help sources emphasise the importance of “finding meaning and purpose” or “renourishing your soul” in ED recovery (Costin and Schubert Grabb, 2011; Ward and Crouch, 2025). While such expressions of spirituality are becoming more accepted in healthcare within Western contexts, religiosity is at times viewed less favourably (Hill et al., 2000; Baumsteiger and Chenneville, 2015). Moreover, some studies have shown that healthcare professionals are less likely than their patients to identify as religious (Koenig et al., 1991; van Nieuw Amerongen-Meeuse et al., 2018). Religiously affiliated individuals have reported experiences of their faith being excluded or minimised in mental healthcare, leading to a sense of disconnection, self-censorship and disengagement (van Nieuw Amerongen-Meeuse et al., 2018; Islam and Chadwick, 2025). As a result, some authors have argued that in some groups, it is not possible to provide person-centred healthcare without considering religious identity (Jabeen and Snowden, 2022).

In the Australian and New Zealand context, there has been a recent shift away from majority Christian affiliation, with an increase in people identifying with “no religion” (Australian Bureau of Statistics, 2022b). While Christianity remained the most frequent religious affiliation in Australia in the 2021 census (43.9%), this was declining, particularly among younger people (Australian Bureau of Statistics, 2022b). In New Zealand, “no religion” (51.5%) comprised the majority in the 2023 census (Stats NZ, 2024). Most previous research related to religion, spirituality and EDs has been conducted in the USA, where Christianity comprises a majority (62%) of the population and religion is increasingly thought to be gaining societal influence (Alper and Sandstrom, 2025; Rotolo, 2025). To our knowledge, there are no previous studies exploring this topic in the increasingly secular Australian or New Zealand contexts. We have chosen to focus on Christian religion and spirituality in this study due to its ongoing relevance to a significant portion of this population, and because several authors bring a lived experience of Christianity to the research. Christian religion can be understood as the beliefs, experiences and practices endorsed by Christian tradition, and Christian spirituality as a person’s sense of relation with the God revealed in Jesus Christ, which influences their relations with others, themselves, and nature (Holder, 2005; Ruffing, 2005; Scorgie, 2011).

This paper reports one dimension of a larger study that aims to explore experiences and perspectives of multiple affected groups (including people with lived experience of an ED, carers/close supports, healthcare providers and Christian pastoral carers) in Australia and New Zealand regarding Christian religion and spirituality in ED development, experience, care and recovery. This larger study seeks to co-create a resource to support the acknowledgement, addressing and/or integration of Christian spirituality in ED prevention and care where this is appropriate, desired and likely to enhance care. This paper focuses on lived experience data from people who have a Christian background and have experienced an ED. It reports their experiences and perspectives regarding the interplay between Christian religion and spirituality on the one hand, and ED development, experience and recovery on the other.

## 2 Methods

### 2.1 Design

This study employs a qualitative design, using semi-structured interviews to develop an understanding of relationships between Christian religion and spirituality, and ED experiences. It adopts a critical realist paradigm, which affirms the existence of objective reality alongside acknowledging that our perception of this reality is influenced by individual and sociocultural factors (Bhasker, 2008; Archer et al., 2016; Price and Martin, 2018). This is consistent with a mainstream Christian worldview, which finds its ontological principle in the Christian God, yet acknowledges the limitations of human understanding (Webster, 2012). Data were analysed using reflexive thematic analysis, chosen for its flexibility and ability to stay close to the data while distilling important themes and allowing for researcher interpretation (Braun and Clarke, 2006, 2021a). To enhance depth of analysis, we also chose to employ some grounded theory techniques (line-by-line coding, concurrent data collection and analysis), consistent with the suitability of such techniques to explore topics with limited existing research (Charmaz, 2006). Findings are reported according to the Consolidated Criteria for Reporting Qualitative Research (COREQ) guidelines (Tong et al., 2007).

### 2.2 Study setting

The study was conducted with people living in Australia or New Zealand. EDs are increasing in prevalence in these regions, with an Australian lifetime prevalence estimated at 10.45% (Chadwick, 2024; Deloitte Access Economics, 2024). Australia and New Zealand are high income constitutional democracies, whose founding documents prohibit establishment of a national religion, and enshrine religious freedom (Section 116, 1901; Sections 13-15, 1990; Lab, 2025). This is reflected in the diverse religious and spiritual affiliations of their people, as discussed above. Both Australia and New Zealand have Indigenous populations (Aboriginal and Torres Strait Islander and Māori respectively) with histories of traditional forms of spirituality (Keane, 2025; Australian Institute of Health and Welfare, 2025).

### 2.3 Ethics statement

Ethics approval was obtained from the University of Queensland’s human research ethics committee (2023/HE001764). Participants received information about the study aim and procedures and provided written informed consent. Confidentiality was maintained through de-identification of responses in interview transcripts and research outputs. Measures to mitigate the risk of causing distress or triggering ED symptoms were implemented, including a requirement that participants self-identified as at least partially recovered from their ED, and were able to identify personal support structures. Interviewers were responsive to any signs of participant distress, and participants were free to withdraw from the study at any time prior to data analysis.

### 2.4 Study participants

This paper reports results from the ED lived experience subset of a larger study which also examined the experiences of carers/close supports of people with EDs, and healthcare professionals, Christian leaders, and pastoral carers serving people with EDs. Eligibility criteria for ED lived experience participants included self-reporting having experienced an ED; living in Australia or New Zealand at the time of the study; current or prior Christian affiliation or exposure to Christian contexts; age 16 years or older; and English language fluency. Exclusion criteria included absence of self-identified support structures, current hospital admission and acute psychosis.

Participants were recruited through purposive and snowball sampling. The study invitation was disseminated through Christian media, social media, authors’ institutional websites, seminar presentations, relevant professional organisations and authors’ personal contacts. Researchers did not seek to establish a relationship with participants prior to study commencement, although some participants were known to members of the research team.

### 2.5 Data collection

Participants completed a short online survey (Supplementary File 1) which was developed and distributed using Qualtrics survey software (Qualtrics, Provo, UT). This survey was used to confirm that participants met inclusion criteria, inform purposive selection of interview participants and provide the interviewer with background information to guide interviews. As there were more survey respondents than were required for interviews, respondents who met inclusion and exclusion criteria were purposively invited to an interview, based on demographic diversity. Specifically, we aimed for a sample of diverse age, gender, geographic location, religious affiliation, and type of ED.

Selected participants were invited to an individual, online, semi-structured interview with a member of the research team (HT or CO), using *Zoom*. ED lived experience interviews were conducted between January 2024 and May 2025 and lasted 50–101 min (average 75). The interview schedule was modified iteratively during data collection and analysis to explore emerging themes (Supplementary File 2). Participant responses were summarised and confirmed during the interview; interview transcripts were not sent to participants for checking due to existing critiques of this approach as a validity check, and for feasibility reasons (Motulsky, 2021). Some participants volunteered additional information via email. Data collection continued until researchers considered that adequate information power (i.e., sufficient richness and quality of data to achieve the study aim) was obtained (Malterud et al., 2016; Braun and Clarke, 2021b). Interviews were video-recorded and transcribed using a professional transcription service, then de-identified and imported into *NVivo 14* (Lumivero, version 14.23) for analysis.

### 2.6 Data analysis

Transcripts were analysed using reflexive thematic analysis (Braun and Clarke, 2006, 2021a), together with some grounded theory techniques (line-by-line coding, concurrent data collection and analysis) (Charmaz, 2006; O’Callaghan et al., 2024). Initially, HT familiarised herself with the data by producing a written narrative summary of each transcript. She manually performed initial line-by-line reflexive coding of transcripts; both semantic and latent coding were performed when appropriate (Byrne, 2022). A second researcher (CO) read all transcripts, reviewed HT’s codes, and annotated interview transcripts in *NVivo* (Lumivero, version 14.23). The annotations noted areas of agreement, suggested additional codes, and stated different views about the coded text where relevant. HT reviewed CO’s annotations, and either accepted these or engaged in further discussion until both were satisfied with the coding structure. Such discussion aimed to enrich analysis through considering diverse perspectives (Kitto et al., 2008; O’Callaghan et al., 2024). NS and MBe read selected transcripts, and NS provided written theoretical memos to HT. Themes and subthemes were produced by HT and discussed among the research team until all analysts were satisfied that they accurately represented the research data.

### 2.7 Trustworthiness

Several procedures supported trustworthiness of the research findings. In-depth interviews were conducted to enable the interviewer to build rapport with participants and deeply understand their perspectives. The interviewer made field notes immediately following each interview, and a reflexive process of qualitative analysis was used to stay close to the data. Researchers sought to be reflexive, discussing their personal positionings and seeking to remain aware of their influence during data analysis (see Supplementary File 3 for further information about authors’ backgrounds). The research team represented a range of disciplines (general practise, psychology, theology, palliative care, ethics, social work and music therapy), male and female genders, and inclusion of members who did and did not profess Christian belief. This diversity supported investigator triangulation, through review of all codes by a second analyst (CO), and discussion of themes among the research team.

## 3 Results

### 3.1 Participant characteristics

Seventy-three complete, non-duplicate, ED lived experience survey responses were received to May 2025. Ten were excluded as they did not meet eligibility criteria [not at least partially recovered (*n* = 9) or not living in Australia or New Zealand (*n* = 1)]. Twenty-six eligible ED lived experience participants were selected for demographic diversity (see section 2.5 above) and invited to an interview, and 23 accepted (three did not arrange an interview time). Eight of these interviewees belonged to more than one participant group—that is, they identified as a carer, healthcare provider and/or pastoral carer in addition to having ED lived experience. Although the remaining 37 survey respondents were not invited to interview, many of these participants have indicated their willingness to be involved in future stages of this research.

Lived experience interviewee ages ranged from 18 to 57 years (average 34 years, excluding one participant who did not specify her age). One participant identified as male, with the remainder identifying as female. The majority were from Australia (82.6%) and identified as Caucasian or European (65.2%). Twenty participants (87%) identified as Christian (representing a variety of denominational backgrounds as well as non-practising Christians), while three (13%) identified as either atheist, agnostic, or spiritual but not religious. All participants who did not report their ethnicity as Caucasian or European identified as Christian, with a variety of denominational affiliations. Many participants reported multiple ED diagnoses. Diagnoses reported included anorexia nervosa (60.9%), bulimia nervosa (30.4%), binge eating disorder (17.4%), other specified feeding and eating disorder (21.7%) and rumination disorder (4.3%). Two participants (8.7%) self-reported avoidant-restrictive food intake disorder, however their description of their ED during the interview seemed more consistent with anorexia nervosa or atypical anorexia nervosa. Most participants (56.5%) self-identified as partially recovered from their ED, with the remainder identifying as fully recovered. For further detail regarding interviewee demographics, see Supplementary File 4.

### 3.2 Thematic analysis findings

The overarching theme resulting from analysis of ED lived experience data conceptualised both EDs and participants’ spirituality as prolonged, dynamic and non-linear journeys, which were often deeply intertwined. Most lived experience participants felt that Christian religion or spirituality had influenced their ED experience, though views on the nature of its impact were mixed.

Three themes, each with two to three subthemes, expand upon this dynamic (Table 1). Participants’ deep sense of being “not enough” often underpinned their ED, and some believed that this was exacerbated by their experience of religious perfectionism and/or spiritual abuse. For most participants, their ED was associated with religious and spiritual “wrestling,” which could lead to a change in their spiritual trajectory. Those participants who identified as Christian at the time of the interview often reported that their Christianity provided them with “help to heal,” though for some its impact was mixed.

**Table 1:** Themes.

Theme	Subthemes	Illustrative quotations
1. “Not enough”	“Not good enough”	*‘It’s…like your appearance is the outward showing that you are a good person, if you are not overweight, if you are fit, if you eat healthy food…That judgement around food choices for me is very linked to…the only way you can be a good Christian is if you have a perfect life and nothing ever goes wrong…all your ducks in a row…you are financially stable, you are emotionally stable…It always felt to me like something you had to work for. And I know the grace bit, and I get it. But even though I get it, and I understand it, that was not the day-to-day working…within Christian organisations’ (LH14).* *‘I do not know how familiar you are with…the idea of purity culture or that kind of concept…It was…very influenced in terms of…what is considered an appropriate…shape, body size, appearance…The idea of being good, very much entrenched with that discipline and that control’ (LH89).*
	“Not safe enough”	*‘I think I have grown up with a lot of rules that I set up for myself. Yes. I mean obviously, at times people would say them directly to me, but a lot of them were…like, “You have to do this to be safe.” And…I would never have articulated it that…concretely. But…the way it would show up is…when I had chronic fatigue, I was like, “Well, if you are going to be a safe, good Christian, you need to be at church every week. You need to be at Bible study every week. You need to be serving in ministry. You need to be praying this much. You need to be reading the Bible every day. And if you don’t do those things, then…God is not happy with you and he’ll be disappointed in you, and he will withdraw from you”’ (L9).* *‘My family got involved in a church that was very harmful. It was a cult…We were actually in that for a few years. I went through a lot of trauma with that’ (L11).*
2. “Wrestling”	Spiritual struggle	*‘That first six months of university was a big faith wrestle for me because I was like, “Is this real? Is God real? I’m in so much pain right now. Does he have anything to say about that?”…I was having all these questions about God. Reading my Bible would make me even more anxious. Because I’d be reading my Bible and I’d be like, “Oh, maybe this isn’t real. Maybe my whole life is built on something that’s not real.” And so, it was a, a huge existential wrestle in that time…I really did that wrestling, that…“This is so awful. Where are you? You know, this is so dark”’ (L9).* *‘Definitely…I think one of the biggest issues for me especially when I had my binging…and…obviously purging episodes was…the guilt I felt…There were elements where I just thought… [God] must be so sick of me, I keep on saying this, “I am so sorry, I am so sorry.” You know, I am in tears…and then…the cycle happens again…I kept on saying, “No, no. No, okay, I am going to stop, I am going to stop”…It was just a massive cycle to (L46).*
	Spiritual transformation or disengagement	‘*At like 10 [years old] or just before 11-ish…is when I kind of just stopped caring [about Christianity], paying attention.* *Interviewer: Yeah, about the same time - - -* *Yeah.* *as becoming unwell’ (P50).* *‘I went to…Sunday school as a kid, and then I turned into an agnostic, and I remember a Jewish family sending me a Bible when I was a teenager. I remember sending it back with a super rude note…It’s not for another…2 years after that to I’m on my knees and my life is a complete mess that I’m calling out to God’ (LCHP10).* *‘I wouldn’t be where I am today if [God] just took [the ED] from me. Like, I do not think I would have learnt as much. I wouldn’t have been strengthened so much. My faith wouldn’t have been as…deep as it was’ (LH53).*
3. Help to heal	Created good and embraced by grace	*‘I’m fearfully and wonderfully made in the image of God, and I accept myself with unconditional love’ (L42).* *‘[Jesus] took our sins, so gave us His grace…This probably speaks to me the most. At the same time, we are more wicked than we could ever believe, but we are more loved than we could ever imagine’ (LCP17).*
	“My safe place”	*‘[God] was my safe place…and he would hide me…Because I felt like I was just being attacked so much…in my dreams…with words…by people, things like that. So, I had to hide in Him and He would be my safe place’ (LH53).* *‘[God’s unconditional love]…just helps with…believing in yourself, I guess. Once you are…getting better…leaving [the ED] behind, if that makes sense. And…the idea that you have always got a place and someone who loves me’ (L67).*
	Hope and empowerment	*‘For me, [suffering well] looks like continuing to run to Jesus all the time in my suffering…And believing that he is the answer, and my ultimate way that I’m going to get through it. And so, what that has looked like is, yes, I go and pursue professional help…I go and see people that are helpful…But my context is we live in a broken, fallen world. Crap happens, and this is part of that. And it won’t be forever. And that…gives me hope that it’s not forever. We’re not alone…And [God is] going to create meaning out of what we are going through. And somehow, some way it is making me more like Jesus, and it’s for my good. And I think if I did not have that, I couldn’t do it. I would—I would—I do not know what I’d be doing’ (L9).* *‘[God’s] resources never run out…He created the world by just speaking…it’s okay to be so desperate and relying on him’ (LH54).*

While the concepts of religion and spirituality are theoretically distinguishable as discussed previously (section 1), our participants tended to refer to “Christianity” or “Christian faith” during their interviews, in a way that could encompass one or both aspects. We have reflected participants’ voices by generally adopting this more encompassing language when reporting results. Non-specific terms (e.g., “a small number,” “some,” “several,” “many”) are used at times to provide some indication of how common described views and experiences were within this sample. Specific numbers are not used except to describe the sample’s demographic characteristics, as the study’s qualitative methodology was not designed to precisely determine the frequency of experiences or themes/subthemes among the sample, or for any such frequency data to be generalisable (Neale et al., 2014). Finally, throughout this paper, participant quotations are coded “L,” denoting ED lived experience. To identify the eight participants who also identified as a carer (C), healthcare provider (H), and/or pastoral carer (P), the relevant combination of codes is used (e.g., LH = lived experience and healthcare provider).

#### 3.2.1 “Not enough”

Participants emphasised that their EDs were not simply about food and body image, but rather a manifestation of deep distress. One explained:

*‘This is one of the most important things I’d like people to know about eating disorders—The expression of an ED, and their symptoms of behaviours to change weight and shape represent many things that are much deeper and related to self-worth & value. Bad body image and ‘feeling fat’ is the manifestation of paralysing shame, feeling unlovable, insecure, isolated, like I’ll never be accepted, like I’ll never accept myself. I feel like I’ll never be taken seriously, unhappy, scared and that everything is out of control. Unfortunately, the messages that society sends us today are around body shape equalling worth, and a myriad of expectations around ‘milestones’ in life. This makes changing my body feel like the most accessible and tangible way to fix those thoughts’* (L87).

Their stories included multiple known risk factors for EDs, including biological (e.g., family history, dieting, neurodiversity), developmental (e.g., family dynamics, adverse childhood experiences), psychological (e.g., perfectionistic traits, emotional repression, depression, anxiety, stress), relational (e.g., difficulty in interpersonal relationships, experiences of trauma and abuse) and/or sociocultural (e.g., societal attitudes to food and weight) factors (Barakat et al., 2023). Additionally, some identified religious and spiritual factors that they believed had contributed, as outlined below. Participants explained that these diverse factors interplayed to create a sense that they were “not enough”; that is, “not good enough,” “not safe enough”, or both. In this context, their ED often subconsciously offered a “*little haven*” (L46) through control, emotional suppression, avoidance of fears or a sense of identity and empowerment.

##### 3.2.1.1 “Not good enough”

Several participants expressed that a deep sense of not being “good enough” underpinned their ED. One had believed, “I’m not good enough and I don’t deserve good things” (LH78), and another, “felt that there was something intrinsically wrong with me” (L87). For some, this included a painful sense that they were not a “*good Christian*” (L9).

Participants believed that a sense of not being good enough could be exacerbated by religious perfectionism. Several participants described childhood experiences in which they learned to understand Christianity in terms of being “good” and engaging in religious service. This was sometimes accompanied by a sense that “I needed to be all these things that I wasn’t” (L11). For some, this understanding arose within rigid family contexts. One described an “enormous lack of autonomy” in her childhood, which she explained was understood as “a Christian thing, that you were out of control of your life and God controlled you. But that’s because my Mum controlled me and she was kind of putting herself in the space of God” (L9). Additionally, some had worked in Christian organisations that they perceived to have unrealistic expectations of employees’ performance, implying that performing to an “exceptionally high standard” was evidence of “*genuine faith*” (LH14). Participants reflected that people with perfectionistic tendencies, who were thereby at increased risk of developing EDs, could be particularly sensitive to these dynamics. One explained:

*‘I’ve got enough pressure in my own head around perfectionism…I just don’t need the added pressure of a church laying over the top...I think those two things just collided in a church space of…no matter how hard you try, you’re never actually going to reach perfection in either the eating disorder or Christianity…I think for me, it was like, “All right, well, if I can’t do it in both, I’ll just go hammer for tongs to do it in one of those”’* (LH14).

Additionally, participants believed that when Christian “goodness” was understood primarily in terms of self-discipline, control, restraint and self-sacrifice, this could enable restrictive EDs in those at risk. One shared that her ED was initially unrecognised due to fasting being “lauded… perceived as a more faithful person” (LH89) in her childhood church context. She described this context as being defined by “*purity culture*,” which she felt placed responsibility upon women to “protect...the men in your life” from sexual sin by not being “too feminine in your appearance.” She believed that this contributed to her ED, having reflected that “I may not be able to change the shape [of my body], but at least I can keep it smaller rather than more pronounced” (LH89). This dynamic was intensified when Christians perhaps unconsciously appropriated societal ideals concerning food and weight, thereby giving them additional moral force.

In these contexts, several participants expressed a deep sense of shame for falling short of what they perceived to be God’s standard. They expressed that they had “no problem hearing that I was a bad sinner…I was like…I’m a bad, bad, child” (L9), and felt that they were “a disappointment…not worthy of God’s love” (L15).

##### 3.2.1.2 “Not safe enough”

Alongside this sense of not being “good enough,” several participants identified that a deep sense of being unsafe underpinned their ED. One explained: “People are not safe…and eating disorder…is available…it’s not out of reach” (LCHP10). Another described using food to dissociate from an embodied sense of being unsafe: “my body was very uncomfortable all the time, and eating was what I did to numb that” (L9).

This sense of lacking safety often arose from experiences of childhood trauma or abuse, though could also be triggered by situations that disrupted participants’ sense of stability, such as moving out of home, reproductive transitions, relationship breakdowns and fear of the future. Religious and spiritual aspects could interplay with these factors in several ways.

One participant described experiencing a loss of identity, purpose and stability when she began to question her childhood faith. She explained that this existential distress contributed to her ED onset:

*‘I didn’t have an anchor, so the ED became that anchor…I didn’t have a specific faith anymore or…groundedness to…talk to God and rely on Him…So…the ED was like, “Yes, I will ground you and make you feel better and look after you and keep you safe”’*. (L35)

Some participants who had grown up in contexts of religious perfectionism reflected that they had acquired a deeply embedded fear of God’s judgement, sensing that they were only a “*safe Christian*” if they kept religious rules (L9). One identified that she projected her experience of her mother onto God, an experience she described as being characterised by “control, manipulation, disappointment…if I ever did the wrong thing I’m punished, I’m shamed” (L9).

Additionally, in some contexts of religious perfectionism, mental health struggles were denied or stigmatised. Some participants described an underlying belief that Christians do not, or should not, experience mental health issues, associated with a culture of emotional repression and judgement. One described an attitude among some Christians that, “You should pray more…If you’re a real Christian, then you wouldn’t have a mental health issue…the reason you have this is that you must have sin” (LCHP10). Several others described similar experiences, some having experienced ostracisation by their faith communities due to mental illness.

Moreover, experiences of religious or spiritual trauma and abuse exacerbated some participants’ EDs. For example, one described financial manipulation, believing that a Christian employer had tried to make herself and her colleagues feel like *“terrible heathens”* for receiving a wage rather than working “for the love of God” (LH14). Another reported sexual abuse perpetrated by religious community members, though not by clergy in this study. One participant explicitly linked her ED with a sense of disempowerment within Christian contexts, explaining: “I was so unwell and so disempowered…[it] reinforced that…I can’t say anything in this space, but I can continue to run further and restrict food. It’s like…what can I control?” (LH14) Others expressed that they felt unable to individuate due to a perceived lack of autonomy in Christian contexts, a sense that they were not allowed to question their faith, and a reliance on their Christian community for a sense of identity.

#### 3.2.2 “Wrestling”

Despite promising security, participants’ EDs became increasingly consuming and controlling, resulting in physical compromise, disrupted relationships, impaired ability to fulfil their usual roles and inner *“turmoil”* (L15). They described a sense of being “trapped and fully consumed” as “the war against my body, and ultimately myself, spiralled exceedingly quickly” (L87). One reflected, “you lose everything, even your ability to laugh, because you have no energy…I couldn’t do anything. I was silent” (LCP17).

This suffering represented a significant stressor, that frequently prompted spiritual struggle and often led to a shift in how the person understood or engaged with religion and spirituality.

##### 3.2.2.1 Religious and spiritual struggle

For many participants who identified as Christian at its onset, their ED prompted significant religious and spiritual struggle. This could manifest as intrapsychic, divine and/or interpersonal struggle.

At an intrapsychic level, participants frequently described a deep sense of guilt and shame for experiencing an ED. This could relate to feeling that their ED was a result of something that they had or had not done, to experiencing “*dark thoughts*” (P67), or to the negative impacts of their ED (such as causing harm to their body, causing their loved ones distress, and impeding their ability to serve God). Many experienced a deep sense of guilt for causing harm to their body, yet felt stuck and unable to change, explaining:

*“I felt guilty because God created me in His image…it’s the temple of the Holy Spirit, the body. And I felt like I was just disrespecting God and throwing it back in His face…the dichotomy…of wanting to please God and loving Him so deeply…But at the same time feeling so guilty because…I’m sort of just letting myself whittle away and…if this gets worse, I could die…It was awful”* (L15).

Conversely, a small number of participants reported that knowing that the ED “is not good for me and God wouldn’t like it” (LH34) helpfully prompted behavioural change.

Additionally, many participants reported experiencing divine struggle during their ED. This could involve anger with God, feeling let down by God, or questioning God’s goodness, control, or existence in the face of suffering.

Many participants also described interpersonal religious and spiritual struggle during their ED. Several described “a funny juxtaposition” in that while “on a personal level, God felt very close and present…in a corporate context it felt very…distance creating and shame inducing” (LH89). Several described experiences of stigma and judgement for having an ED, which they related to their faith community’s primarily spiritual understanding of the condition (attributing EDs to sin, lack of faith, or demonic influence). One resigned her employment with a Christian organisation and chose to stop attending church (while maintaining her Christian faith) after her workplace maintained that her ED hospitalisation was a consequence of her sin, and questioned her spiritual competency to fulfil her work role. Less overtly, some participants felt disappointed that their faith community did not provide the support that they had expected throughout their ED journey.

##### 3.2.2.2 Spiritual transformation or disengagement

The experience of an ED and associated struggle often influenced participants’ religious and spiritual trajectories.

Three participants in this study did not identify as Christian at the onset of their ED. For them, struggles around the time of their ED prompted their conversion to Christianity. One described agreeing to attend church with a family member, where:

*‘I closed my eyes and I said, “God, if you're real, show me.” And then in that instant, the Holy Spirit just filled me and I just cried…It just all came out and it just felt like God was…washing me clean…It was just so much healing, but hurt as well of, like, what I had gone through on my own…So that was how…I met Jesus’* (LH53).

The remaining twenty participants reported a Christian upbringing or conversion prior to their ED. Their religious/spiritual journeys took a variety of directions following the onset of their ED, with some explaining that their position was still evolving. Several explained that the all-consuming nature of the ED led to caring less about, or reducing participation in, Christian practices. Some described avoiding practices involving food (including taking communion). Alongside this, despite experiencing religious and spiritual struggle, multiple participants *“lean[ed] into”* (LC49) their faith in response to their ED (see section 3.2.3). Some developed a new perception of God’s stance towards them, with a deeper understanding and experience of God’s grace. They described this as deeply healing, though often continued to describe a tension between their understanding of God’s grace and judgement. Some participants went through a period in which they no longer identified as Christian amidst questioning why God would allow them to suffer, before later re-engaging (often in response to the persistent love of their Christian family or community) or moving to a less conservative Christianity. Some who had grown up in what they described as *“high control”* (LH89) Christian contexts withdrew from Christian community, while retaining personal Christian faith, in order to remove themselves from a context that they believed contributed to heightened perfectionism, and/or to mitigate the risk of religious trauma. One referred to her ongoing process of religious *“deconstruction”* (LH89) and described her church attendance as “fairly hit and miss,” related to caution arising from previous experiences. The three participants who did not identify as Christian at the time of the study had gradually moved away from their Christian upbringing as they transitioned to adulthood, concluding that Christianity was not “right for me anymore” (L11). Two of these identified as atheist or agnostic at the time of the interview, while another described ongoing questioning and movement towards a more generic spirituality. She described this process of *“questioning everything”* as having “almost helped me,” as she experienced relief from an internalised pressure to have all the answers and rather learned to “[hold] it all gently” (L35).

#### 3.2.3 Help to heal

Many participants who identified as Christian at the time of the interview believed that despite struggling with faith, their Christianity supported their journey towards whole person recovery. The degree to which they believed that it did so varied, with some describing it as entirely or primarily helpful, while others described a more mixed impact, and a small number felt that it did not have an influence for them, as they had not considered the relevance of their faith to their ED until after they had recovered. This variation in perceived helpfulness was present both among participants who identified as fully recovered from their ED at the time of the interview and those who did not.

Participants who found Christianity helpful shared that it could support recovery through deeply addressing their sense of being “not enough,” and through offering hope and empowerment.

##### 3.2.3.1 Created good and embraced by grace

Several Christian participants shared that a deepened experience of God’s grace engaged their sense of not being “good enough” and provided a foundation for healthy identity.

Participants explained that Christian teaching that all people are “fearfully and wonderfully made, in the image of God” (L42) provided a basis for their own dignity and value. Alongside this, they were often painfully conscious of the Christian doctrine of sin. One shared, “I had no problem hearing I was a bad sinner. That was not hard for me…to accept at all…I was like…I’m a bad, bad child...But understanding grace, or understanding belovedness—what is that?” (L9) This concept of *“grace”* and “*belovedness”* was echoed by multiple participants, who reported finding these deeply healing, particularly in struggles with perfectionism, guilt and shame. Many understood grace to be God’s unconditional acceptance and love based not on personal worthiness or performance but rather on that of Jesus: “*We are more wicked than we could ever believe, but we are more loved than we could ever imagine*…*completely and utterly acceptable before God, because…of what Jesus has done.*” (LCP17) As a result, they acknowledged that “[sin] is not…our identity, it is not who we are” (L54), but rather, “We are a child of God…we are loved…we are precious…we were bought at a cost.” (LH78).

Participants shared that grace-based identity supported ED recovery through providing a basis to extend love and grace towards oneself, supplanting a quest for perfection through rule-keeping. They explained that, “We have to learn to love ourselves or accept ourselves right there…because Jesus does” (LCP17). It also helped some to reframe a perceived connection between their appearance and their personal value. One shared:

‘*My value and worth wasn’t based on my body image, it wasn’t based on my weight, it wasn’t based on even my performance or my academic ability or anything like that. It was all because of…how [God] saw me and how He created me’* (LH53).

Participants identified that embracing—or being embraced by—grace was often an ongoing process. Several who grew up in Christian contexts reflected that they were not “aware of experiencing grace until much later” (LH14), and that “Maybe [I’d] never known [unconditional love]…it was really profound” (L9). They experienced grace and love through various avenues, including deeply moving internal experiences of God’s grace amidst suffering, spending time prayerfully reflecting on related Christian teaching and Scripture, and through the embodiment of grace in other people. Yet several shared that internalising grace was an ongoing challenge; one shared, “I have lots of grace for other people. I have only judgement for myself” (LH14).

##### 3.2.3.2 “My safe place”

When participants were able to internalise God’s grace and love, several shared that this could alleviate their sense of not being “safe enough.” It helped to counter a reliance on rules to provide safety; one participant reflected, “They’re just rules that I’ve made up…actually…what keeps me a safe Christian is Jesus” (L9). For some participants, trusting God’s presence with them provided a safe place, in contrast to the false safety of the ED:

*‘The main [Scripture] that I remember now is “I’ll never leave you nor forsake you.” Because I think that through my life, lots of people have left me…and have forsaken me when I needed help the most…[God] was my safe place…and He would hide me’* (LH53).

This belief that God was with them and understood the complexity of their struggle was comforting for many participants. Such security enabled them to “[wrestle] through stuff that really hurts and not run away from it” (L9). One shared how a sense of God’s supportive presence enabled her to begin to eat more consistently, explaining:

*‘With the eating disorder you feel compelled to…hide your food or eat the least amount when no nurse is watching…but just knowing God was always there, He was always watching…it helped calm the eating disorder down a little bit…I just felt His presence…like a light…a good presence rather than the presence of the eating disorder is quite tormenting and controlling. So, I could tap into, or feel, or sense that presence rather than just the presence of the eating disorder’* (L54).

Additionally, several participants reported experiencing safe faith community relationships. One felt safe to eat with her church small group, as “I’m in this really safe environment with people who really care about me and…if anything [emotion] spilled out because I was too overwhelmed, they wouldn’t be weird about it” (L43).

This sense of safety and basis for identity helped to create a supportive scaffolding for participants to risk change and growth.

##### 3.2.3.3 Hope and empowerment

Participants shared that ED recovery was a long, non-linear and painful journey. One Christian with a long-standing ED poignantly described having given up hope of recovery. Others shared that for them, their Christianity fostered hope. For some this represented hope for recovery, and for others, hope that regardless of the outcome God would use their suffering for good. One explained: “[God’s] going to create meaning out of what we’re going through. And somehow, some way, it is making me more like Jesus and it’s for my good. And I think if I didn’t have that, I couldn’t do it” (L9).

For several participants, Christian faith provided motivation to recover. This often entailed a sense of purpose. A participant who had moved from Christianity to more generic spirituality shared a similar sentiment to some Christian participants, that she was “here for a reason and…created for a purpose…I’m here for more than the ED…there’s meaning and purpose to my life and I can make a difference” (L35). Several participants believed that part of their purpose was to support others experiencing an ED, having been equipped by their own experience, and some who had recovered were doing so in a variety of ways.

Several Christian participants also believed that God helped them to recover. One described that her ED caused her to be “desperate [for] and relying on [God]” (LH53). Such reliance took various forms. Some participants prayed for healing and viewed their ED as “a problem I…left for [God] to handle” (LP40). Others prayed for help to do what was required each day, expressing a belief that they could not “do things…by my own strength” but needed God’s *“help and…wisdom”* (L15). This could involve active surrender: “I hand myself over to you…you’re in control” (LH15), though this participant acknowledged that while she desired such surrender, it was often short-lived. Alongside this, some participants described a very collaborative process of recovering with God. One shared:

*‘All the times that…I was involved in all the bulimia and…all the crazy behaviour that goes with it, never once did I hear the Holy Spirit say to me…you’re an idiot, you didn’t do good. I never heard it, ever. The things I heard were, “It probably wasn’t your best choice, LCHP10, let’s try again”…He would always just be…“We can do this.” We, we, it was always about we’* (LCHP10).

Several participants shared that their Christian beliefs equipped them to challenge ED cognitions. Some conceptualised this as an internal *“spiritual battle”* (LH78), in which they fought against ED beliefs by challenging them with Scripture. Some explained that viewing their body as God’s good creation helped them to accept, trust and care for it, recognising that diversity in body shapes and sizes was intended by God. Similarly, one participant’s belief that all foods were provided by God and therefore “not inherently bad” helped her to “be a bit more flexible in my approach” (L65). Another was able to reframe eating as a “costly act of worship,” in response to a verse that encouraged “taking the everyday acts of your life and them being an act of worship” (LH89, likely referring to Romans 12:1). Some explained that they memorised Scripture and helpful quotations and repeated them to themselves when they were struggling, such as when they experienced an internal sense of condemnation or their self-worth was “*attacked”* (LH53) by others’ words. They explained that this provided a “reference point to what is valid, what is real, what is true…I needed a solid concept to…hold onto” (L54).

Several recovered participants believed that their experience of Christianity enabled deep healing, supporting “change…in my heart…how I saw myself” rather than simply “more symptom management or behavioural change” (LH53). One summarised, “one of my catch phrases in life is…‘mercy kissed me’ *because I just feel that’s true…I have just been kissed by mercy and…my whole life has been rebuilt*” (LCHP10).

## 4 Discussion

Our findings highlight the intertwining of participants’ EDs and their religious and spiritual journeys. Participants described a complex and bidirectional influence, whereby aspects of Christian religion and spirituality could both exacerbate and alleviate EDs, and EDs could influence individuals’ religious and spiritual trajectories. The influence of Christianity on EDs could be conceptualised primarily as exacerbating or alleviating a person’s deep sense of not being “good enough” or “safe enough,” and offering a potential source of empowerment and healing.

These findings draw on existing theories related to religious coping and the impact of mental health struggles on spiritual journeys (Pargament, 2007, 2010), together with those suggesting the importance of a “sense of safety,” mediated by complex biological, psycho-neuro-immunological and social mechanisms, in mental wellbeing. The results are consistent with previous studies that suggest a mixed association between religion, spirituality and EDs (Akrawi et al., 2015; Richards et al., 2018; Blair, 2019). They also align with previously identified predisposing and protective factors for EDs, such as perfectionism (Robinson and Wade, 2021), and Bruch’s early theorising regarding the role of attachment and self-concept in anorexia nervosa (Treasure and Cardi, 2017). Our study suggests an additional and often overlooked aspect of such constructs in our participants—namely, that they can encompass a religious and/or spiritual domain which may be deeply important to the person and interact with their ED both helpfully and unhelpfully. The development of new or modified approaches to treatment that incorporate religious and spiritual aspects, based on an understanding of individuals’ religious and spiritual beliefs and backgrounds, may be indicated.

Participants’ sense of not being “good enough” reflects the known link between EDs, perfectionism and low self-esteem (Wade et al., 2015; Krauss et al., 2023; Stackpole et al., 2023). However, participants’ perfectionism extended beyond traditional domains to encompass a sense of not being a “good enough Christian.” The under-recognition of perfectionism applied to a religious domain is reflected in religion’s absence from a list of 22 “domains of perfectionism” (Stoeber and Stoeber, 2009; Wang et al., 2020). Like accepted understandings of perfectionism, “religious perfectionism” can be conceptualised as a multidimensional construct comprised of perfectionistic strivings (“zealous religious concern”) and perfectionistic concerns (“religious self-criticism”) (Stoeber and Otto, 2006; Wang et al., 2018; Wang et al., 2020). It should be noted that this is not necessarily synonymous with the theological concept of “Christian perfection”, which describes a process of spiritual maturing in love through the enabling of God’s Spirit, rather than an infallible state (Campbell, 2013).

Multiple participants emphasised a perceived link between perfectionism applied to Christianity and their ED experience. Some described family rigidity, which has previously been found to correlate with “religious dysfunctional perfectionism” in an Australian sample (Craddock et al., 2010). Religious perfectionism could indirectly influence ED onset, or act more directly through an unbalanced emphasis upon self-denial and self-sacrifice, or the amalgamation of Western cultural health and body image ideals into one’s concept of being a “good Christian”. Quantitative investigation to confirm such a relationship may be worthwhile, particularly due to its potential clinical relevance. Well-evidenced cognitive-behavioural treatments for perfectionism in EDs are available (Robinson and Wade, 2021); these could potentially be adapted to incorporate relevant religious dimensions.

Some participants distanced themselves from their previous Christian upbringing or affiliation during their ED and recovery, explaining that this no longer felt right for them, or that they needed to remove themselves from a context that exacerbated their perfectionism or put them at risk of experiencing trauma. This distancing was often a gradual process, involving questioning, uncertainty and redefining personal identity, meaning and purpose. This unsettling process could create existential distress and interact with participants’ EDs both helpfully and unhelpfully, yet was often unrecognised in ED therapy. This highlights a need for therapists to be alert to the potential of religious or spiritual existential distress in EDs, to acknowledge it when present, and to offer non-judgemental support as people navigate their way through these issues.

Conversely, many participants retained their Christian affiliation during their ED and recovery. Among these, a strong theme was that understanding and internalising God’s grace could be experienced as deeply healing. Participants’ understanding of grace aligned with that in previous research as, “the unmerited expression of God’s love, in which God offers the gift of relationship with Godself” (Hall and McMinn, 2021). This understanding of grace does not necessarily negate high religious standards and expectations. However, internalising grace could help to alleviate perfectionism by providing assurance that God’s compassion, acceptance and love towards oneself was not contingent upon one’s achievements. This could inform treatments for Christian perfectionism that aim to foster a religious identity that is not dependent upon achieving perfectionistic standards, and to encourage self-compassion, which can be understood as mirroring God’s grace towards oneself. Such approaches could help to address participants’ frequent ongoing dialectic between an intellectual understanding of God’s grace and their difficulty experiencing it personally, which has also been identified in other contexts (Hodge et al., 2022; Snow et al., 2023).

Participants’ sense of not being “safe enough” aligns with the link between EDs, trauma and insecure attachment, expressed in our participants’ embodied sense of “unsafety” (Molendijk et al., 2017; Trottier and MacDonald, 2017; Jewell et al., 2023; Lynch et al., 2025). Again, religious dimensions of these constructs are under-recognised. Experiences of trauma could impact participants’ perception of and attachment to God. God can function as an attachment figure (Cherniak et al., 2021), being described in the Bible in parental terms (e.g., Matthew 6:9; Isaiah 66:13). Insecure (anxious, and sometimes avoidant) attachment to God has previously been associated with ED symptoms (Homan and Boyatzis, 2010; Exline et al., 2016; Strenger, 2016; Fannon and Goodman, 2025). These findings align with the experience of participants in our study. Alongside this, learning to understand and experience God as gracious and unconditionally loving, together with an assurance that God would never leave them, were viewed by several as key to healing. Secure attachment to God could provide a “safe haven” offering comfort and hope during participants’ EDs, together with a “secure base” from which to risk change. This is consistent with previous research suggesting that grace can provide Christians with a source of strength, resilience and growth and is inversely correlated with psychological distress (Bronte and Wade, 2012; Hyde and Joseph, 2022; Snow et al., 2023). It could represent an additional resource in ED therapies informed by attachment theory. For example, the voice of an abusive parent could be replaced with the loving voice of God in imagery rescripting (Arntz, 2025). Christian contemplative traditions may also represent a novel treatment adjunct, representing scope for future work (Timbers and Hollenberger, 2022).

Finally, some participants described experiences of religious and spiritual abuse, which has been defined as “a form of emotional and psychological abuse characterised by a systematic pattern of coercive and controlling behaviour in a religious context or with a religious rationale” (Oakley et al., 2024). The findings are consistent with other studies in which such abuse represented a self-reported risk factor for EDs, though research is limited (Crocker, 2021; Pooler and Droesch, 2025). As mainstream discussions of abuse tend to focus on its physical, psychological/emotional and sexual manifestations, experiences of religious and spiritual abuse in EDs may be overlooked, representing a missed opportunity for support (Caslini et al., 2016; Pignatelli et al., 2017). This represents an area for future research.

Strengths of this study include its multidisciplinary research team, who bring a range of perspectives and backgrounds to analysis, and its diverse participant demographics and Christian denominational affiliations. The results add to the limited existing literature concerning religion, spirituality and EDs and is to our knowledge the first qualitative study exploring this topic in the Australian/New Zealand context. It highlights the relevance of religious manifestations of known ED risk factors, such as religious perfectionism and religious and spiritual abuse, which are currently under-recognised and at risk of becoming increasingly so with growing societal secularisation. Additionally, it suggests avenues that could be explored in adapting ED care for Christians, such as considering grace-based identity and fostering secure attachment to God.

Limitations include an over-representation of female participants. This was due to difficulty recruiting participants of non-female gender, perhaps due to the overrepresentation of EDs among females and associated stigma. There were a majority of Caucasian or European participants, however this is broadly reflective of Australian demographics—while Australia represents a multicultural community, a majority of respondents reported European ancestry in the 2021 census (Australian Bureau of Statistics, 2022a). Indigenous participants were under-represented, and findings may not reflect their experience. Many study participants had self-reported comorbid mental health conditions alongside their EDs, which may have impacted their religious and spiritual experience. It is not always possible to differentiate between the impact of their EDs and these other conditions. However, given the high rates of mental health comorbidity in EDs, this is reflective of a real-world context (Hambleton et al., 2022). Additionally, further research would be needed to confirm any differences between participants related to specific ED diagnosis or Christian denominational affiliation. There were insufficient participant numbers in this study to confidently compare affiliations. This was particularly the case as many participants’ denominational affiliations were fluid, and several preferred to identify simply as a Christian who currently attended a church of a particular denomination than to adopt a denominational identity. Additionally, this paper touches only briefly on the clinical implications of study findings. We hope to further develop these in a future publication that incorporates carer and healthcare provider perspectives.

Finally, our study focused specifically on Christian religion and spirituality. This enabled identification of aspects that may be specific to this faith tradition and ensures that interventions developed from the results are likely to be salient to this group, rather than potentially too broad to be helpful. However, it implies that results may not be transferable to people with other religious or spiritual backgrounds. Notably, for example, grace has been identified as uniquely emphasised within the Christian tradition (Hall and McMinn, 2021). Further research is required with other groups to identify points of similarity and difference to inform approaches to care, especially within multi-cultural and multi-religious communities.

## 5 Conclusion

This study highlights the intertwined nature of EDs and Christian spirituality for our many of our participants, encompassing both helpful and unhelpful impacts. Christian religion and spirituality interacted with participants’ ED journeys, influencing their sense of being “enough” (“good enough,” “safe enough”) through its impact on constructs such as perfectionism, attachment, identity, existential distress and hope.

Findings are informing the next stage of the study, which aims to co-design a resource for clinicians, Christian faith communities, carers and people with lived experience of EDs to support ED prevention and care where engagement with Christian religion and spirituality is relevant and desired.

## Data Availability

The datasets presented in this article are not readily available because interview transcripts include sensitive and confidential information. Themes with selected de-identified quotations are available on the Open Science Framework Thomas, H., Best, M., Kimber, T., Sturman, N., Wade, T., Brautigam, M., & O’Callaghan, C. (2026, February 2). Data Sharing: Eating Disorders and Christian Spirituality Project Themes with Selected Deidentified Quotations. https://doi.org/10.17605/OSF.IO/TSR7Y. Requests to access the datasets should be directed to Hayley Thomas, h.thomas@uq.edu.au.
