# Supplementary material for: Christian religion and spirituality in eating disorder development, experience, and recovery: an exploration of lived experience in Australia and New Zealand
Source: Front Psychol. 2026 Feb 17;17:1764418. doi: 10.3389/fpsyg.2026.1764418 (PMC12955360; doi:10.3389/fpsyg.2026.1764418)
Supplement: Supplementary file 1 [file Data_Sheet_1.pdf]

Additional File One: Eating Disorder Lived Experience Participant Initial Survey

Thank you for agreeing to participate in this research. We would appreciate it if you could take the time to complete the following questionnaire about yourself and your experience with eating disorders.

What is your name (please include first name and surname)?

____

What is the best method to contact you regarding participation in this study? Please provide details (eg. email address, phone number) below.

____

Which of the following groups do you belong to (you may select more than one):

- ☐ Eating disorder lived experience
- ☐ Carer or close personal support of someone with an eating disorder
- ☐ Healthcare provider
- ☐ Christian pastoral carer (eg. clergy, church pastoral care team member, chaplain, teacher/well-being coordinator in Christian educational setting)

What is your age (in years)?

- ○ Please enter age below: ____
- ○ Prefer not to say

What is your gender?

- ◯ Male
- ◯ Female
- ◯ Non-binary
- ◯ Prefer not to say

What is your ethnicity?
Please be as specific as possible - for example, if you identify as Australian, please specify 'Caucasian (or European) Australian', 'Asian Australian', 'Aboriginal Australian' etc.

____

What is your current postcode?

____

What is the highest level of education that you have completed?

- ◯ Primary school
- ◯ Secondary school (to year 10)
- ◯ Secondary school (to year 12)
- ◯ Professional qualification, certificate, or diploma
- ◯ Bachelor's degree
- ◯ Graduate certificate or diploma
- ◯ Master's degree
- ◯ Doctoral degree

What is your current occupation (or most recent occupation if you have retired or are on leave)?

____

Please indicate how strongly you agree or disagree with the following statements:

	Strongly Disagree	Disagree	Neutral	Agree	Strongly Agree
I consider myself to be religious	○	○	○	○	○
I consider myself to be spiritual	○	○	○	○	○

What is your current religious affiliation/s?

☐ Christian (please specify denomination, if relevant):________________________

☐ Other religion (please specify):____

☐ No religion (please specify atheist (do not believe in god/s); agnostic (believe nothing can be known of whether god/s exist); spiritual but not religious; and/or other, if relevant):____

☐ Other (please specify): ____

The following questions ask for some basic information about your eating disorder experience. If you are comfortable, we would appreciate your responses.

What is/was your eating disorder diagnosis? If you have been diagnosed with more than one eating disorder in the past, please select all diagnoses.

- ☐ Anorexia nervosa
- ☐ Bulimia nervosa
- ☐ Binge eating disorder
- ☐ Avoidant/Restrictive Food Intake Disorder
- ☐ Pica
- ☐ Rumination Disorder
- ☐ Other Specified Feeding and Eating Disorder (e.g., atypical anorexia nervosa)
- ☐ Other (please specify):____

How old do you think you were when your eating disorder symptoms started?

____

How old were you when you were first formally diagnosed with an eating disorder?

____

What eating disorder treatments or supports have you accessed (please select all that apply)?

- ☐ Support from family members and/or friends
- ☐ Support from religious community and/or spiritual leader
- ☐ Guided self-help resources
- ☐ Books (please specify): _______________________________________

- ☐ Websites (please specify): ____
- ☐ General Practitioner
- ☐ Psychotherapy (e.g., psychologist, counselling) (not during a hospital admission)
- ☐ Twelve Step Groups
- ☐ Dietetics (not during a hospital admission)
- ☐ Exercise physiology (not during a hospital admission)
- ☐ Peer support groups (not during a hospital admission)
- ☐ Eating disorder support organisations (e.g., Butterfly Foundation)
- ☐ Lived experience coach, mentor, or recovery navigator
- ☐ Eating disorder day program
- ☐ Eating disorder residential program
- ☐ Mental health admission
- ☐ Medical admission
- ☐ Other (please specify): ______________________________________

Please list any eating disorder treatment that you have received in the past two years?

____

Please list any eating disorder treatment that you are currently receiving?

____

Currently, would you consider yourself to have recovered from your eating disorder/s?

- I have not recovered
- I have partially recovered
- I have fully recovered

What support structures do you have in place if you were to experience a difficult time in your recovery?

____
