# Supplementary material for: Christian religion and spirituality in eating disorder development, experience, and recovery: an exploration of lived experience in Australia and New Zealand
Source: Front Psychol. 2026 Feb 17;17:1764418. doi: 10.3389/fpsyg.2026.1764418 (PMC12955360; doi:10.3389/fpsyg.2026.1764418)
Supplement: Supplementary file 2 [file Data_Sheet_2.pdf]

Additional File 2: Eating Disorder (ED) Lived Experience Interview Guide

Interviewer to initially:

- Introduce themselves (disclose religious affiliation if asked)
- Explain the interview's purpose and expected duration
- Reinforce information about confidentiality and its limits
- Reinforce that the participant is free to withdraw from the interview at any time
- Re-confirm the participant's consent to record the interview
- Clarify whether the participant has any questions, provide any extra information required, and confirm that they are happy to proceed

Could you tell me about yourself?

- Include probes to explore the participant's ED journey
    - Examples include:
        - Can you tell me about your ED journey?
        - What ED symptoms/behaviours have you experienced? What diagnosis/diagnoses have been made (and when)?
        - What factors do you think contributed to developing your ED?
        - When and how did you realise that you were suffering from ED symptoms?
        - How did you initially access professional help for your ED?
        - What treatment have you received for your ED? Was it helpful, and why/why not?
        - What other supports or resources did you access? Were these helpful or unhelpful, and why?
        - How would you describe your current relationship with food, eating and your body?
- Include probes to explore participants' religious and/or spiritual experience.
    - Examples include:
        - Can you tell me about your religious or spiritual life journey so far? Are there any moments or events that particularly stand out for you?
        - Do you believe in God? If so, how do you perceive God?
        - What are your experiences with religious leaders/ people/ institutions?
        - What are your beliefs about and experience with religious practices?

Could you tell me about whether religion and/or spirituality has influenced your ED journey, and if so, how?

- Include probes to explore:
    - The relevance of religion and spirituality at different stages of their ED, including development, experience, and recovery
    - Helpful and unhelpful aspects of religion and spirituality in their ED journey (Christian religion and spirituality specifically)
    - Beliefs about food/eating/bodies
    - Beliefs about the self/identity
    - Whether members of their faith community were part of their ED journey and whether their input was helpful or unhelpful.

What are your preferences and experiences regarding integrating religious and spiritual aspects in ED treatment?

- Include probes to explore:
    - Experiences of and preferences regarding religious and spiritual integration in treatment
    - Any effect of the degree of alignment between their own religious and spiritual views and those of health professionals upon treatment.

What (if anything) would you like faith communities (e.g., churches, religious schools) to do/not do to help people experiencing EDs?

Examples of Questions that Were Added Iteratively During Data Collection and Analysis:

- Probe understanding of:
    - Grace
    - How the participant conceptualises EDs and whether/how spirituality is relevant to this (e.g. views of 'spiritual warfare', the demonic)
    - What do you believe is God's role, and what do you believe is your role, in ED recovery?
- Explore experiences of/thoughts about:
    - Religious/spiritual trauma
    - Family aspects
        - Were there any family aspects related to religion and spirituality that were helpful or unhelpful in your ED journey?
    - Perfectionism in religious contexts exacerbating ED
        - Did you feel a pressure to be perfect in church settings, and if so, how did this influence your ED journey?
        - Explore perspective on personality vulnerability contribution vs environmental contribution
    - Re-evaluating faith
        - [If faith has been reevaluated in ED journey] What influenced your decision to continue in/move away from your faith?
- Some questions tailored to participants' background, based on data collected in the initial Qualtrics survey; E.g. Do you think that your ethnic background influenced the interplay between your faith and ED?
