# Supplementary material for: Christian religion and spirituality in eating disorder development, experience, and recovery: an exploration of lived experience in Australia and New Zealand
Source: Front Psychol. 2026 Feb 17;17:1764418. doi: 10.3389/fpsyg.2026.1764418 (PMC12955360; doi:10.3389/fpsyg.2026.1764418)
Supplement: Supplementary file 3 [file Data_Sheet_3.pdf]

Additional File Three: Information Regarding Authors' Backgrounds

Dr Hayley Thomas is white, female general practitioner and PhD candidate. She works part-time clinically at a private general practice, where she cares for patients with a broad range of conditions, including some affected by eating disorders (EDs). She also works part-time in academic general practice, with a research interest in whole person approaches to care and the doctor-patient relationship. Dr Thomas identifies as Christian and has been an active member of Protestant churches for most of her life. She has completed a Graduate Diploma of Christian Studies through the Australian College of Theology.

Dr Clare O'Callaghan is a white female who works as a music therapist in palliative care and as a researcher on varied healthcare projects. Throughout her 40-year career, Clare has led and collaborated on many qualitative and mixed methods studies focussed on improving support and outcomes for people affected by life-threatening conditions. Clare has a Roman Catholic background and was once actively involved in church music. Clare currently does not identify as a Christian.

Professor Megan Best is a white, female palliative care physician and Senior Research Fellow. She has an interest and expertise in researching spirituality in healthcare, along with Christian healthcare ethics. Professor Best identifies as a Christian and attends a Protestant Church. She has completed a ATh through the Australian College of Theology.

Reverend Dr Michael Bräutigam is a white, male theologian, pastor, and lecturer, also trained in psychology. He is committed to integrating Christian theology and psychology, in order to equip Christian pastors and professionals to foster mental wellbeing and grow healthy communities, and founded The Centre for Theology and Psychology at the Melbourne School of Theology. Reverend Dr Bräutigam has worked academically in both psychology and theology and in diverse cultural contexts, including Germany (his original home), Scotland and Australia. Michael is an ordained minister with the Free Church of Scotland.

Dr Thomas Kimber is a white, male theologian, who identifies as a Protestant Christian. He is a former Dean of Faculty, Director of Research and Lecturer in Missional and Pastoral Theology at the Melbourne School of Theology. Originally from Southern California and having previously served as a missionary in China, Dr Kimber has a particular interest in the integration of spiritual formation, pastoral theology and mission.

Matthew Flinders Distinguished Professor Tracey Wade is a white, female clinical psychologist and the Director of the Flinders University Services for Eating Disorders. Professor Wade has over 30 years' experience working as a clinician with EDs and has research interests in ED aetiology, prevention, treatment, and research translation, having cowritten four therapy books and published over 300 peer-reviewed journal articles. She holds multiple leadership roles in the ED field. Professor Wade identifies as Christian and has been an active member of Protestant churches for most of her life.

Associate Professor Nancy Sturman is a white, female general practitioner, who works in clinical and academic general practice. She has expertise in medical education, and a clinical focus on health and social care for vulnerable populations. She is familiar with Anglican beliefs and practices, and has worked with non-government organisations auspiced by various Christian churches, but does not identify as Christian.
