# Supplementary material for: Christian religion and spirituality in eating disorder development, experience, and recovery: an exploration of lived experience in Australia and New Zealand
Source: Front Psychol. 2026 Feb 17;17:1764418. doi: 10.3389/fpsyg.2026.1764418 (PMC12955360; doi:10.3389/fpsyg.2026.1764418)
Supplement: Supplementary file 4 [file Data_Sheet_4.pdf]

Additional File 4:
Eating Disorder (ED) Lived Experience Interview Participant Demographics (n = 23)

	n	%
**Participant group***		
Lived experience only	14	60.9%
Also carer/personal support	3	13.0%
Also healthcare provider	2	8.7%
Also pastoral carer	3	13.0%
**Gender**		
Male	1	4.3%
Female	22	95.7%
**Age (Years)**		
16-20	2	8.7%
21-30	6	26.1%
31-40	9	39.1%
41-50	3	13.0%
51-60	3	13.0%
Over 60	0	0.0%
**Ethnicity****		
Caucasian/European	15	65.2%
Other (Aboriginal Australian, Asian, Black, unspecified Australian)	8	34.8%%

**Education**		
Secondary school (to year 12)	3	13.0%
Professional qualification, certificate or diploma	6	26.1%
Bachelor’s degree	6	26.1%
Graduate Certificate or Diploma	1	4.3%
Master’s degree	5	21.7%
Doctoral degree	2	8.7%
**Religious Affiliation or Current Denomination Attended**		
Christian:	20	87.0%
Catholic	*4*	*17.4%*
Pentecostal/Charismatic Protestant	*5*	*21.7%*
Other Protestant	*7*	*34.8%*
Other (non-denominational, non-practising, not further specified)	*4*	*21.7%*
No religious affiliation	3	13.0%
**Religiosity *(‘I consider myself to be religious’)***		
Strongly disagree	0	0.0%
Disagree	4	17.4%
Neutral	3	13.0%
Agree	9	39.1%
Strongly agree	7	30.4%

**Spirituality *('I consider myself to be spiritual')***		
Strongly disagree	1	4.3%
Disagree	1	4.3%
Neutral	2	8.7%
Agree	9	39.1%
Strongly agree	10	43.5%
**ED Diagnosis (Self-Reported)*** **		
Anorexia nervosa	14	60.9%
Bulimia nervosa	7	30.4%
Binge eating disorder	4	17.4%
Avoidant/restrictive food intake disorder	2	8.7%
Other specified feeding and eating disorder	5	21.7%
Rumination disorder	1	4.3%
**ED Recovery (Self-Reported)**		
Not yet recovered	0	0.0%
Partially recovered	13	56.5%
Fully recovered	10	43.5%

Abbreviations: ED – Eating disorder
**Multiple participants identified with more than one participant group*
***Aggregated from self-report*
****Self-reported ED diagnosis. Please note that several participants reported multiple diagnoses. Also, participants who self-reported avoidant/restrictive food intake disorder described symptoms more suggestive of anorexia nervosa in interviews, so avoidant/restrictive food intake disorder participant numbers may be lower, and anorexia nervosa (or atypical anorexia nervosa) higher than indicated.*
